# Is the implementation of smoke-free policies at workplaces associated with living in a smoke-free home?: Findings from a national population-based study in Malaysia

**DOI:** 10.18332/tid/100692

**Published:** 2019-06-07

**Authors:** Kuang Hock Lim, Hui Li Lim, Chien Huey Teh, Sumarni Mohd Ghazali, Chee Cheong Kee, Pei Pei Heng, Rafiza Shaharuddin, Jia Hui Lim

**Affiliations:** 1 Institute for Medical Research, Ministry of Health, Kuala Lumpur, Malaysia; 2 Hospital Sultan Haji Ahmad Shah, Temerloh, Malaysia; 3 School of Science, Monash University Malaysia, Subang Jaya, Malaysia

**Keywords:** secondhand smoke, smoke-free home, smoke-free workplace, population-based study, Global Adult Tobacco Survey–Malaysia

## Abstract

**INTRODUCTION:**

Studies have shown that the implementation of smoke-free policies at workplaces have shifted the social norms towards secondhand smoke (SHS) exposure at home. This study aimed to investigate whether working in a smoke-free workplace is associated with living in a smoke-free home (SFH).

**METHODS:**

The data were derived from the Malaysian Global Adult Tobacco Survey (GATS-M), collected in 2011–2012, involving 4250 respondents. Data analyses involved 1343 respondents reported to be in the working population.

**RESULTS:**

More than half of the respondents (58.5%) were reportedly working in smoke-free workplaces. Almost a quarter (24.8%) of those who worked in smoke-free workplaces stayed in smoke-free homes, which was more than two times higher than their counterparts who worked at non-smoke-free workplaces (24.8% vs 12.0%, p<0.001). Multivariable analyses further substantiated this finding (AOR=2.01, 95% CI: 1.11–3.61, reference group = worked at non-smoke-free workplaces).

**CONCLUSIONS:**

This study found an association between living in smoke-free homes and working at smoke-free workplaces, which could suggest a positive impact of implementing smoke-free workplaces.

## INTRODUCTION

A multitude of national surveys over the last four decades have revealed that the smoking habit is a common behaviour among Malaysian adults. Nearly half of the Malaysian adult males are smokers^1-5^, and this high prevanence of smoking might play a substantial role in increasing secondhand smoke (SHS) exposure in our communities^6^. The National Health and Morbidity Survey (NHMS) 2015 reported that more than one-third of Malaysians aged 15 years and older were exposed to SHS at home and workplace^6^, with the exposure higher among males, rural dwellers, the younger age group, and those with lower educational attainment. Studies have documented that the exposure to SHS is linked to coronary heart disease, respiratory infections and asthma, as well as a variety of cancers^7-9^. Also, it increases the risk of smoking initiation among non-smoking adolescents^10^ and reduces the likelihood of smoking cessation among youth smokers^11^. Recognition of such threats has led the Malaysian government to initiate various policies and control measures to address this problem. The Ministry of Health Malaysia had introduced the expansion of smoke-free zones in public localities and working areas since 1993^12^. Smoking is prohibited at health facilities since 1993, followed by an expansion of smoke-free areas to all government premises, schools and education facilities, including financial institutions such as banks, National Telecom company, National Energy company, and post offices. Smoking is prohibited only on floors with service counters, implying that smoking is permitted on floors with offices but without service counters (provided those floors do not have centralized air-conditioning). In 2009, smoking was prohibited in all workplaces with a centralized air-conditioning system^13^. Also, indoor workplaces located in smoke-free initiatives areas such as the ‘Melaka Bebas Asap Rokok’ (Melaka smoke-free initiatives) had also been restricted^14^. The implementation of the smoke-free workplace was supported by frequent visits of enforcement officers to the abovementioned working areas. Furthermore, the National Institute of Occupational Safety and Health, Malaysia, also issued the guidelines of non-smoking in offices to all employers to enhance indoor air quality in workplaces^15^. However, smoking is still permitted in many types of indoor workplaces, which is not stipulated in the Control of Tobacco Product Regulations.

Various studies have demonstrated a positive relationship between the implementation of smoke-free policies at workplaces and living in smoke-free homes in both developed and developing countries. Kaleta et al.^16^ and Lee et al.^17^ reported an adjusted odds ratio (AOR) of 5.3 and 2.07, respectively, in their studies among the Nigerian and Indian populations who worked in smoke-free workplaces and stayed in smoke-free homes, compared to those who worked in places that allowed smoking. Nazar et al.^18^ completed a study in 15 Lower-Middle-Income Countries (LMIC), which also reported identical positive findings with a pool AOR of 1.61. Apart from the significant association between smoke-free policies and living in smoke-free homes, there is also a significant association between living in smoke-free homes and the smoking status of respondents^16,18^, their education levels^16,18^, the residential areas^16,18^, genders^16,18^, types of occupation^18^, and age groups^16,18^. Although a plethora of studies has been carried out to determine the association and causality factors between working in the smoke-free environment and living in smoke-free homes, there was no such study in Malaysia. Therefore, an investigation of the association between working in smoke-free areas and living in smoke-free homes was needed to provide scientific evidence to motivate public health authorities to formulate suitable policies to prohibit smoking in more indoor working areas in Malaysia. This paper aimed to address these gaps and to generate constructive findings to assist health authorities in proposing a comprehensive smoke-free policy in this country.

## METHODS

This study used data from the Malaysian Global Adult Tobacco Survey (GATS-M), which was carried out in Malaysia from 2011 to 2012. The nationwide study of GATS-M employed a cross-sectional study design and multi-stage proportionate to size sampling method to select a representative sample of non-institutionalized respondents aged 15 years and older. Our survey in Malaysia adopted the methodology and tools suggested in the GATS survey. GATS-M has an overall response rate of 83.1% (n=4250/5112). The sample size of 5112 was based on GATS sampling protocol (in which a sample of at least of 4000 respondents is required; 2000 males and 2000 females with 2000 adults each from the urban and the rural areas). The sample size was then adjusted upward to allow for potential ineligibility and non-response rate. The data obtained were weighted taking into account the complex study design and non-response rate based on the 2010 National Population Census data to ensure its national representativeness. Only respondents who reported to be working within the last 12 months before their interview and working indoors were included in the analysis.

The dependent variable in the study is ‘living in smoke-free homes’, which was measured by an item ‘has anyone smoked inside their home in the past 30 days’. The respondents who answered ‘no’ were classified as ‘living in smoke-free homes’ and ‘yes’ as ‘living in non-smoke-free homes’. The independent variable is ‘working at smoke-free workplaces’. The categorization of smoke-free policy at working areas was classified as ‘total restriction’ (smoking is not allowed anywhere in the building) ‘partial restriction’ (smoking is allowed in certain places in the building) and ‘non-restriction’ (smoking is allowed everywhere and/or policy to restrict smoking in the working areas) was based on individual response, whether their working areas had such restrictions. In addition, smoking status and sociodemographic variables in this study include age group (15–24, 25–44, 45–64, ≥65 years), gender, residence (rural, urban), current use of tobacco products (current smoker, current non-smoker), education level (below primary school level, below secondary school level, completed secondary and higher secondary school, college/university and above), occupation (government sector, private sector or self-employed), and marital status (married, single, widow(er)/divorced), which were all significantly associated with living in smoke-free homes in previous studies^16,18^.

### Statistical analysis

Data cleaning was performed before analysis. The sample was weighted to represent the general population aged 15 years and older, based on the 2010 Malaysia Population Census, study design and response rate of the study. Descriptive statistics were employed to describe the sociodemographic characteristics of respondents while chi-squared analysis was used to investigate the association between the proportion of respondents who lived in smoke-free homes and working in smoke-free areas, and sociodemographic variables (age, gender, residence, marital status, education attainment, occupation, and smoking status). All univariate analyses with p-values less than 0.25 or those significantly associated with living in smoke-free homes from previous studies were included in the multiple logistic regression model (MLR). Two-way interaction between working in the smoke-free environment and smoking status, working in the smoke-free environment and all sociodemographic variables (age, gender, residence, education attainment, occupation) was carried out to determine possible interaction between the independent variables. A p-value of >0.05 indicates that there is no significant interaction between the independent variables. All statistical analyses were carried out at 95% CI using SPSS statistical software (complex sample design) version 22.

## RESULTS

A total of 1140 respondents who had worked for 12 months and indoors were included in the analyses. From the 1140 respondents, about two-thirds of the respondents were males (59.0%). More than three-quarters of the respondents resided in urban areas (80.2%) and were of age <45 years (80.2%). More than half (56.6%) of the respondents reported that they were working in smoke-free areas and less than one-fifth (18.8%) of the respondents reported that they were living in smoke-free homes. Nearly one-third (28.9%) of respondents were current smokers (Table 1).

**Table 1:** Sociodemographic characteristics of respondents aged 15 years and older reporting working in the last 30 days

*Variable*	*Estimated population*	*Sample*	*%*
**Smoking restriction at working areas**			
None	700607	135	11.3
Partial	1990207	345	32.1
Total	3517087	632	56.6
**Living in smoke-free home**			
Yes	639182	124	18.8
No	2767464	489	81.2
**Gender**			
Male	3819459	668	59.0
Female	2649238	416	41.0
**Residential**			
Urban	5186797	726	80.2
Rural	1281901	416	19.8
**Ethnicity**			
Malay	4017473	703	62.1
Chinese	1281888	208	19.8
Indian	602815	82	9.3
Others	566520	148	9.8
**Age group**			
15–24	1428212	168	22.1
25–44	3761182	691	58.1
45–64	3564736	268	19.0
≥65 years	1603529	14	0.8
**Education level**			
Less than primary school	115180	34	1.8
Less than secondary school	1148017	207	17.9
Completed secondary or high school	3564735	629	55.4
College and above	1603529	269	24.9
**Occupation**			
Government	1383699	303	22.0
Private	4007207	653	63.6
Self-employed	905448	168	14.4
**Income level**			
Quintile 5	2248135	349	35.1
Quintile 4	1633201	301	25.5
Quintile 3	1348017	228	21.2
Quintile 2	751033	148	11.9
Quintile 1	410762	101	6.4
**Marital status**			
Married	3862405	742	59.8
Single	2409918	373	37.3
Widow(er)/divorced	190051	60	2.9
**Smoking status**			
Yes	1904566	330	28.4
No	4564131	811	70.6

The proportion of respondents living in smoke-free homes was slightly higher among respondents who reported to be working under partial and total restriction of smoking in workplaces in comparison to respondents whose workplaces allowed smoking practices. Besides, the proportion of respondents living in smoke-free homes was also found to be higher among females, respondents residing in rural areas, of older age groups, those with higher education attainment, higher income quintile groups and current smokers. Multivariable logistic regression demonstrated that the odds of living in smoke-free homes was 2 times higher among respondents who were working in partial and total smoke-free environments compared to those who were working in non-smoke-free areas (total smoking restriction, AOR=2.93; 95% CI: 1.20–7.16; partial restriction, AOR=2.58, 95% CI: 1.05–6.31, with non-restriction as the reference). Non-current smokers (AOR=32.26, 95% CI: 15.36–66.66) and females (AOR=2.75; 95% CI: 1.41–4.35) were more likely to live in smoke-free homes than current smokers and males (Table 2).

**Table 2:** Prevalence and factors associated with living in a smoke-free home among Malaysian working adults – percentages and adjusted odds ratios (AORs) are weighted

*Variable*	*Living in smoke-free home*				*Factors associated with living in smoke-free home*	

	*Estimated population*	Sample^*^	*%*	*95% CI*	AOR**	*95% CI*
**Smoking restriction at working areas**						
None	75249	15	16.9	9.3–28.6	Ref.	Ref.
Partial	245226	41	19.9	13.7–28.0	2.58	1.05–6.31
Total	292725	64	19.1	14.0–25.5	2.93	1.20–7.16
**Gender**						
Male	329421	66	15.1	10.9–20.4	Ref.	Ref.
Female	309761	58	25.4	18.9–33.2	2.75	1.41–4.35
**Residential**						
Urban	463139	65	17.6	13.3–22.9	Ref.	Ref.
Rural	176042	59	22.8	16.9–30.0	1.19	0.69–2.06
**Ethnicity**						
Malay	419952	83	18.3	13.8–23.7	0.37	0.17–0.80
Chinese	138655	25	24.7	15.6–36.8	Ref.	Ref.
Indian	56472	9	26.5	11.6–49.0	1.01	0.27–3.84
Others	24102	7	7.2	2.7–18.2	0.36	0.12–1.05
**Age group**						
15–24	133710	16	16.0	8.9–27.2	Ref.	
25–44	353109	76	18.0	13.8–23.1	1.24	0.30–3.28
45–64	152363	32	25.6	16.4–37.7	3.55	2.01–5.28
≥65 years	14615	2	100			
**Education level**						
Less than primary school	5934	2	7.1	1.5–28.3	0.36	0.06–2.28
Less than secondary school	102606	21	16.3	9.1–27.4	0.87	0.27–2.77
Completed secondary or high school	332348	68	17.1	12.7–22.8	1.09	0.57–2.09
College and above	198239	33	27.3	18.8–38.0	Ref.	
**Occupation**						
Government	191221	44	29.6	20.7–40.4	Ref.	
Private	354373	66	16.0	12.1–20.9	0.51	0.25–1.07
Self-employed	75681	12	16.5	8.0–30.9	1.85	0.62–3.53
**Income level**						
Quintile 5	264141	45	23.6	16.9–32.0	Ref.	
Quintile 4	156184	33	20.6	13.2–30.8	1.28	0.65–2.54
Quintile 3	121071	23	15.3	9.4–23.9	0.75	0.35–1.63
Quintile 2	79493	16	17.1	9.6–28.7	0.86	0.32–2.34
Quintile 1	10322	5	4.7	1.6–12.8	0.41	0.12–1.42
**Marital status**						
Married	378956	76	20.6	16.0–26.2	Ref.	
Single	248585	39	16.8	11.3–24.3	1.67	0.83–3.39
Widow(er)/divorced	11641	9	14.0	5.3–31.8	0.68	0.20–2.32
**Smoking status**						
Yes	71726	10	5.0	2.1–11.4	Ref.	
No	567456	114	28.7	23.2–34.8	32.26	15.38–66.66

* The figure for sample only for those who live in a smoke-free home. The number of not living in smoke-free home are: smoking restriction at work (n=474), gender (n=489), residential area (n=489), ethnicity (n=489), age group (n=482), education level (n=485), occupation (n=484), income (n=484), marital status (n=488), and smoking status (n=489).

** Interaction between smoke-free workplace (SFW) x occupation p=0.063; SFW x marital status p=0.197; SFW x wealth index p=0.723; SFW x gender p=0.854; SFW x education attainment p=0.632; SFW x ethnicity p=0.221; SFW x smoking status p=0.622; SFW x locality p=0.093. The Multivariable Logistic Regression analysis was based on an estimated population of 3051980 and sample n=574.

## DISCUSSION

The study found a significant association between the smoke-free legislation in the workplace and living in smoke-free homes in Malaysia. Our finding was consistent with the findings of other studies in other developed or developing countries. The longitudinal studies conducted by Edward et al.^19^ from 2003 to 2006 showed an increase in smoke-free homes from 64% to 70%^19^, in line with the decline of SHS exposure at workplaces from 20% to 8% following the expansion of the smoking ban at workplaces. Fong et al.^20^ also reported a reduction in home smoking rates from 85% to 80% after the implementation of comprehensive smoke-free legislation at workplaces. In addition, comparable positive results were generated from studies by Kaleta et al.^16^ , Lee et al.^17^ and Nazar et al.^18^ among Nigerian, Indian and adults from 15 LMIC countries, respectively, in which respondents living in smoke-free homes were 5.3, 2.07, and 0.6 times, more likely to be among those employed in a totally smoke-free workplace compared to those in non-smoke-free workplaces, after adjustment for potential confounders. Our findings might be explained by the ‘Norm Spreading’ in the life cycle model of social norms, as smoke-free norms developed and created from smoking prohibited workplaces may expand into other localities, more so in homes^17,21,22^. Another plausible reason might be due to those people who are against smoking and whose homes are smoke-free opting for smoke-free workplaces where available. However, future studies are strongly recommended to elucidate the factors contributed to the findings in our current study.

In our study, non-smokers were more likely to live in smoke-free homes. This finding is in line with Kaleta et al.^16^ and Thomas et al.^23^. It may be that non-smokers have a negative attitude towards smoking. This affects their behaviours, such as not allowing this practice in their homes. In addition, the smoking status may create non-permanent norms for other families to smoke in their homes. However, this aspect needs to be investigated in depth in future studies. In contrast to the findings by Nazar et al.^18^, our study found that female respondents were more likely to live in smoke-free homes. The low prevalence of smoking among women in several studies conducted at the national level reduces their risk of SHS exposure. A similar explanation might be applied to the higher likelihood of ethnic Chinese living in smoke-free homes. However, the level of income, education level, marital status and living quarters were found to be insignificant in multivariate analyses after confounder effects were conveyed. These findings contradict the findings by Kaleta et al.^16^ and Berg et al.^24^ This may be due to proxy by education status, which does not lead to behavioural changes, as the health effects of exposure to SHS require a long period of time to show an impact. This aspect needs to be detailed in future studies.

Nevertheless, only 18.8% of the currently working respondents reported to be living in smoke-free homes. This figure was noticeably lower than the prevalence studies reported in China (21%), Thailand (73%), and Mexico (75%)^18^. We postulated that one possible explanation for this lower prevalence in Malaysia was most likely due to smoking being accepted as a normative behaviour among the Malaysian population. Therefore, they have a high tolerance towards smoking and SHS exposure that ultimately leads to widespread recognition of smoking and exposure of others to tobacco smoke as a norm. In addition, the habit of smoking has been accepted by the Malaysian society for ages whilst legislation on smoking restriction in the workplace has only been implemented recently. Thus, this might lead to a low rate of practising it^13^. However, more profound investigation on this aspect should be carried out in future studies. Our findings also suggest that more aggressive measures and strategies are necessary to increase the proportion of smoke-free homes in Malaysia to be in line with the recommendations of Article 8, of the Framework Convention on Tobacco Control (FCTC), which urged all parties to set suitable initiatives to reduce secondhand smoke exposure in public places, working areas, and at home^25^. Future policies should focus on the generation of a smoke-free environment in all workplaces, as our current smoke-free regulation primarily focuses on smoking prohibition only in certain premises such as health care facilities. There is also only one sub-regulation related directly to smoke-free working areas (i.e. smoking is prohibited in all working areas with central air-conditioning systems)^13^.

### Limitations

There are several limitations in this study. Firstly, the status of smoke-free homes and working areas were based on self-reported data. Therefore, it might be under or over reported due to recall bias. Secondly, this current cross-sectional study in which both variables (smoke-free workplaces and smoke-free homes) are measured simultaneously limits the causal interpretation of our findings. For example, we cannot ascertain whether having workplace policies influences the adoption of smoke-free homes or those living in smoke-free homes chose to work in smoke-free workplaces. To address this, a longitudinal study on tobacco use and exposure among Malaysian adults should be carried out^26^.

Lastly, a substantial proportion of our respondents did not respond to an item to measure the dependent variable (i.e. living in smoke-free homes). This might affect the overall results. However, the analysis in this study had controlled for several potential confounding factors including education, gender, smoking status, and geographical location, hence, enhancing the validity of our findings. In addition, our study had a large sample size of the representative sample that enabled the findings to be generalized to the Malaysian population. Furthermore, our study employed the standard protocol and tools recommended in GATS nationwide, allowing a standard comparison with other countries using similar protocols and tools.

## CONCLUSIONS

Our present study demonstrates that the proportion of smoke-free homes reported among working Malaysian adults was small and remained non-prevalent nationwide. Our study suggests that respondents working in smoking-restricted workplaces are more likely to live in smoke-free homes in Malaysia, which can ultimately reduce SHS exposure and improve the health status of the Malaysian population. Therefore, specially-tailored public health policies related to smoke-free environments in working areas are warranted. On the other hand, aggressive voluntary smoking restrictions at home could also potentially be achieved through the expansion of the community-based health promotion and intervention program (KOSPEN – *Komuniti Sihat, Pembina Negara* – Health community, Building the Nation) among the Malaysian community.
